# Dataset on Galanin Receptor 3 mutants that improve recombinant receptor expression and stability in an agonist and antagonist bound form

**DOI:** 10.1016/j.dib.2017.04.057

**Published:** 2017-05-04

**Authors:** Thao T. Ho, Jasmine T. Nguyen, Juping Liu, Pawel Stanczak, Aaron A. Thompson, Yingzhuo G. Yan, Jasmine Chen, Charles K. Allerston, Charles L. Dillard, Hao Xu, Nicholas J. Shoger, Jill S. Cameron, Mark E. Massari, Kathleen Aertgeerts

**Affiliations:** a Department of Structural Biology, Dart Neuroscience, 12278 Scripps Summit Drive, San Diego, CA 92131, USA; b Department of Preclinical Development, Dart Neuroscience, 12278 Scripps Summit Drive, San Diego, CA 92131, USA; c Department of Biology, Dart Neuroscience, 12278 Scripps Summit Drive, San Diego, CA 92131, USA

**Keywords:** Galanin Receptor type 3, GPCR recombinant protein expression, Protein engineering, Virus-like particles, GPCR protein stabilization, Membrane protein

## Abstract

Galanin Receptor 3 (GALR3) is a G-protein-coupled receptor with a widespread distribution in the brain and plays a role in a variety of physiologic processes including cognition/memory, sensory/pain processing, hormone secretion, and feeding behavior. Therefore, GALR3 is considered an attractive CNS drug target (Freimann et al., 2015) [1]. This dataset contains GALR3 point mutants that improve recombinant protein expression and thermal stability of the receptor contained in virus-like particles (VLPs) or obtained by detergent-purification of baculovirus-infected insect cells. The mutations listed can be grouped in those that improve the stability of the agonist-bound and the antagonist-bound form of the receptor. Protein characteristics in terms of protein expression and thermal stability were comparable between GPCR-VLP and GPCR overexpressing Sf9 cultures. The further analysis and detailed results of these mutants as well as their impact on biophysical assay development for drug discovery can be found in “Method for Rapid Optimization of Recombinant GPCR Protein Expression and Stability using Virus-Like Particles” (Ho et al., 2017) [2].

**Specifications Table**

**Table:** 

Subject area	Biology
More specific subject area	GPCR Protein Engineering and Stabilization
Type of data	Table, figure
How data was acquired	Western Blot, Radioligand binding assay, HPLC, thiol-specific fluorochrome N-[4-(7-diethylamino-4-methyl-3-coumarinyl)phenyl]maleimide (CPM) assay [3], Electrospray ionization (ESI)-Mass Spectrometry (MS), hybrid quadrupole time-of-flight mass spectrometer (Q-ToF Ultima, Waters, Manchester, UK)
Data format	Filtered and analyzed
Experimental factors	Does not apply
Experimental features	GALR3 mutants were produced and screened in virus-like particles using label and label-free assay formats; recombinant receptor protein expression and stability was obtained in virus-like particles and after detergent-solubilization from recombinant Sf9 expressions.
Data source location	Does not apply
Data accessibility	The data are included in this article

**Value of the data**

• The GALR3 mutants listed here aid in increasing the recombinant protein expression yield and stabilization of the receptor in either the agonist or antagonist-bound form.
• Mutational analysis of the GALR3 variants was performed in VLPs. The chemically stabilizing environment of the phospholipid bilayer in a VLP eliminates the need for recombinant overexpression, purification and detergent solubilization during the iterative protein engineering process.
• GALR3 protein quality from VLPs was comparable to detergent-purified receptors from overexpressing Sf9 cultures.

## 1 Data

**Fig. 1:**
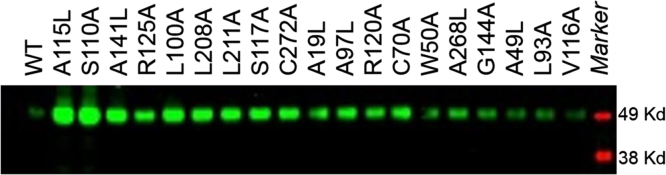
Western blot analysis of GALR3 WT and point mutants. The point mutants were selected out of 210 GALR3 mutants generated using the VLP platform and based on a *B*_max_ (mutant)/*B*_max_ (WT) value of ≥1.0 measured using a [^125^I]-porcine galanin radiometric competition binding assay. The 19 mutants were then expressed in insect cells and lysates were run on an SDS-PAGE. The protein of interest was visualized by Western blot utilizing an anti-6xHis epitope antibody directed against the GALR3 C-terminal 6xHis-tag.

The dataset of this article provides information on GALR3 mutants that stabilize the receptor in either an agonist-bound or antagonist-bound form. Table 1 shows how 23 of the 210 point mutants expressed on VLPs increase [^125^I]-galanin binding and thermal stability compared to WT. In addition, a direct correlation of the *B*_max_ values with recombinant expression yields of the mutants in Sf9 cultures was shown (Table 1 and Fig. 1). Combinations of these mutants were made to find the best thermostabilizing GALR3 agonist-bound (Table 2) and GALR3 antagonist-bound (Table 3) variants.

**Table 1:** Comparison of GALR3-VLP samples to protein samples expressed in Sf9 insect cells.

**GALR3 mutant**	***B***_**max**_**(mutant)/*B***_**max**_**(WT) VLP samples**	**Tm(mutant)-Tm (WT) VLP samples**	**Density (mutant)/Density (WT) Sf9 cell lysate**
WT	1.0	0	1.0
A115L	7.0	2.9	6.7
S110A	5.3	5.6	6.4
A141L	3.8	3.3	4.4
R125A	3.2	3.7	3.1
L100A	3.1	3.7	4.3
L208A	3.0	3.5	3.7
L211A	2.9	3.3	3.6
S117A	2.8	3.3	3.7
C272A	2.3	3.8	3.6
P21A	2.0	2.5	N/A
A19L	1.8	2.2	2.4
A97L	1.8	3.7	3.1
A236L	1.7	1.5	N/A
R120A	1.7	3.3	2.7
C70A	1.7	2.0	3.2
R235A	1.6	3.4	N/A
W50A	1.5	4.0	2.0
A268L	1.5	2.0	2.4
G144A	1.5	1.7	1.8
A49L	1.2	4.4	1.1
L93A	1.1	4.7	1.4
V116A	1.1	6.1	0.8
A198L	1.0	2.3	N/A

Summary of 23 (out of a total of 210) GALR3 mutants that exhibited a *B*_max_ (mutant)/*B*_max_ (WT) value of ≥1.0 with their concomitant thermal stability relative to WT. Both values were obtained from a [^125^I]-porcine galanin radiometric competition binding assay using VLP-GALR3 samples. VLP samples were prepared in a small scale, only sufficient for one thermal stability experiment, performed in duplicate. Protein expression yield improvement relative to WT was obtained consequently of the same mutants when recombinantly expressed using baculovirus-infected Sf9 insect cells. These values are presented in the table as relative band intensities measured from a Western blot analysis utilizing an anti-6xHis epitope antibody directed against the GALR3 C-terminal 6xHis-tag. Small-scale expression samples were used and only sufficient for one Western-blot analysis. The intensity of the protein bands corresponding to the GPCR of interest was measured by the software ImageJ (https://imagej.nih.gov).N/A means not available.

**Table 2:** Protein characteristics of GALR3 agonist-bound mutant combinations.

**GALR3 mutant**	**Total protein yield (µg/L Sf9 culture)**	**SEC peak ratio (%monodisperse/aggregated)**	**Tm (°C)+galanin**
**WT (reference)**	**37**	**10**	48^*^
S110A+V116A	91	90	56
A115L+A198L+C272A	120	83	56
S110A+A115L+L208A	96	76	55
S110A+A115L+R120A	48	50	50
S110A+A115L+L211A	79	62	55
S110A+A115L+A198L	53	62	53
S110A+A115L+A236L	38	50	56
S110A+V116A+A236L	120	93	62
S110A+V116A+R125A	160	90	61
S110A+V116A+L100A	244	86	58
S110A+V116A+S117A	201	87	61
S110A+V116A+L211A	152	81	60
S110A+V116A+C272A	188	76	59
S110A+V116A+L208A	228	93	59
S110A+V116A+A141L	95	95	59
S110A+R120A+A141L+A236L	196	74	53
S110A+V116A+L208A+A236L	183	86	62
S110A+R120A+L208A+A236L	49	92	55
S110A+R120A+A141L+L208A+A236L	169	96	57
S110A+A115L+R120A+A141L+L208A+A236L	97	60	54

Mutants are shown for which ≥50% monodispersity could be achieved after mid-scale (300 ml) recombinant expression in insect cells followed by detergent solubilization and purification. Expression was done once and provided enough material for one Tm measurement. Receptor purity and monodispersity was analyzed using SDS-PAGE and analytical size-exclusion chromatography (SEC). Thermal stability was measured by the thiol-specific fluorochrome N-[4-(7-diethylamino-4-methyl-3-coumarinyl)phenyl]maleimide (CPM) assay [3] *Note that WT GALR3 protein was mostly aggregated and its Tm value cannot be accurately measured. However, GALR3WT was added to the table as a reference.

**Table 3:** Protein characteristics of GALR3 antagonist-bound mutant combinations.

**GALR3 mutant**	**Total protein yield (µg/L Sf9 culture)**	**%Mono-dispersity**	**Tm (°C) (+DNS001131702)**
**WT (reference)**	**44**	**54**	**36**
R120A+A223L	62	89	58
	79	87	42 (+DNS001355175)
V116A+R120A	106	78	51
V116+A223L	82	68	55
V116A+R120A+A223L	72	90	58
	72	90	42 (+DNS001355175)

Mutants are shown for which ≥50% monodispersity could be achieved after mid-scale (300 ml) recombinant expression in insect cells followed by detergent solubilization and purification in the presence of antagonist DNS001131702 or DNS001355175. Expression was done once and provided enough material for one Tm measurement. Thermal stability was measured by the CPM assay [3].

## 2 Experimental design, materials and methods

### 2.1 Characteristics of GALR3 point mutants in VLP versus detergent purified receptors from overexpressing Sf9 cultures

Initial rank ordering of 210 GALR3-VLP point mutants, mostly located in GALR3 transmembrane helices, was based on expression yield obtained from a radiometric assay measuring the binding of [^125^I]-galanin to the receptor and comparing *B*_max_ values of the mutants relative to the *B*_max_ value of WT GALR3. 23 of the 210 mutants showed *B*_max_ (mutant)/*B*_max_ (WT) values of ≥1.0 and an increase in thermal stability relative to WT GALR3 (Table 1). 19 GALR3 point mutants were then recombinantly expressed in Sf9 insect cells to measure protein expression yields as determined by SDS-PAGE/Western blot band intensities (Fig. 1). Expression levels of the 19 single mutants followed the same rank-order as the *B*_max_ values measured by the radiometric assay on the corresponding VLP samples (Fig. 1 and Table 1).

### 2.2 GALR3 agonist-bound thermostabilizing variants

The best GALR3 single point mutants were combined for mid-scale (300 ml) recombinant expression in Sf9 insect cells and protein parameters such as expression yield, monodispersity and thermal stability in the presence of galanin are listed in Table 2, summarizing the best mutant combinations that improved the agonist-bound protein characteristics of the receptor.

### 2.3 GALR3 antagonist-bound thermostabilizing variants

In order to find the stabilizing GALR3 antagonist-bound mutants, we used the data from the GALR3-VLP [^125^I]-galanin binding assay described above to select 88 single point mutants with *B*_max_ values ≥80% of WT, assuming that these displayed enough receptor density to be further evaluated in a size-exclusion chromatography/liquid chromatography mass spectroscopy (SEC/LC-MS) based binding assay. Prior to SEC/LC-MS, the GALR3-VLP samples were incubated with GALR3 antagonist, DNS000135175 (Ki=55 nM) or DNS001131702 (Ki=127 nM) and subjected to a temperature gradient ranging from 15 °C to 55 °C. Before the acquisition of the LC-MS spectra of the ligand, unbound ligand was removed by SEC. A total of 5 single point mutants were found to stabilize the antagonist form of GALR3 by ≥4 °C when compared to the WT form, and rank-ordered from most to least stabilizing as: A223L (Δ*T*=8.3 °C), A218L (Δ*T*=7.2 °C), R120A (Δ*T*=5.4 °C), V116A (Δ*T*=4.6 °C), E224A (Δ*T*=4.2 °C).

Similar to the GALR3 agonist-bound studies, the best GALR3 single point mutants were combined for recombinant expression in Sf9 insect cells and protein parameters such as expression yield, monodispersity and thermal stability in the presence of a GALR3 antagonist-bound form are listed in Table 3.
