# Supplementary material for: Dataset on Galanin Receptor 3 mutants that improve recombinant receptor expression and stability in an agonist and antagonist bound form
Source: Data Brief. 2017 May 4;12:603–7. doi: 10.1016/j.dib.2017.04.057 (PMC5430141; doi:10.1016/j.dib.2017.04.057)
Supplement: Supplementary file 1 — Supplementary material [file mmc1.docx]

**Manuscript Ref**: DIB-D-17-00205

**Manuscript Title**: Dataset on Galanin Receptor 3 mutants that improve recombinant receptor expression and stability in an agonist and antagonist bound

**Authors**: Ho T. *et al.*

Conflicts of interest: none
